# Pre-Workout Supplements and Their Effects on Cardiovascular Health: An Integrative Review

**DOI:** 10.3390/jcdd12040112

**Published:** 2025-03-24

**Authors:** Yanesko Fernandes Bella, Samantha Rodrigues Silva Cupido, Pedro Augusto Querido Inacio, Marcelo Luiz Peixoto Sobral, Rodolfo P. Vieira

**Affiliations:** 1 Department of Medicine, School of Medicine, University Center of the Americas (FAM), São Paulo 01304-001, SP, Brazil; yanesko@hotmail.com (Y.F.B.); samantharscupido@gmail.com (S.R.S.C.); 2 Laboratory of Pulmonary and Exercise Immunology (LABPEI), Evangelical University of Goias (Unievangelica), Anápolis 75083-515, GO, Brazil; pedroqinacio@gmail.com; 3 Department of Cardiovascular and Thoracic Surgery, Heart Institute, University of São Paulo (USP), São Paulo 05508-220, SP, Brazil; mlpsobral@uol.com.br

**Keywords:** heart disease risk factors, dietary supplements, performance-enhancing substances, illicit drugs

## Abstract

Introduction: Dietary supplements have become a popular aid for improving training performance. Pre-workout supplements contain a mixture of ingredients used to boost physical performance, with some components having been associated with the promotion of cardiovascular health. However, there is insufficient scientific literature on the effects of pre-workout supplements, and the studies often have conflicting results. Objective: The aim of this review was to analyze the impact of multi-ingredient pre-workout supplements on cardiovascular health, in order to identify the main adverse effects and the roles of the most common substances in these supplements. Methodology: A systematic search was carried out in the Web of Science and PubMed databases by three independent researchers between January 2010 and August 2024. The inclusion criteria were available articles published in English. Articles that did not evaluate cardiovascular outcomes and the use of pre-workout supplements were excluded. Results: The 24 studies analyzed demonstrated an overall increase in supplement intake. Pre-workout supplements were associated with improved physical performance and possible cardiovascular changes, with these effects being classified as adverse or cardioprotective. This discrepancy in the results may be due to the different dosages and populations investigated (including active and non-active participants, and healthy participants or those with a history of cardiovascular diseases), as well as other factors that correlate with deleterious cardiac conditions. Conclusions: Multi-ingredient pre-workout supplements may offer physical and cardiovascular benefits, including increased energy, focus, endurance, and strength during exercise, as well as having potential positive impacts on blood pressure and triglyceride, low-density lipoprotein (LDL), and homocysteine levels. However, due to the conflicting results of the analyzed studies, additional studies are necessary to fill in the knowledge gaps and establish clearer guidelines for the safe and effective use of these supplements.

## 1. Introduction

A dietary supplement is defined as nutrient, food component, or non-food compound that is consumed with the purpose of achieving specific benefits for performance and/or health [1]. Supplements can be classified as contributing to muscle mass gain or weight loss, improving sports and cognitive performance, or offering convenience (foods in the form of bars or energy drinks) [2,3].

Pre-workout supplements are consumed before physical exercise and often contain a combination of ingredients including caffeine, creatine, and arginine, among others. These supplements are used with the intention of increasing the availability of energy substrates, reducing fatigue, and creating conditions that favor physical performance, endurance, strength, and muscle mass gain [4]. Pre-workout supplement intake can be classified as acute when used for a short period or chronic when its use exceeds 8–12 weeks or is continuous [5].

In addition to their benefits for physical performance, pre-workout supplements have also been studied for their influence on cardiovascular health. Pre-workout supplements often include compounds that can affect the cardiovascular system. These effects can be negative, such as causing increased blood pressure, or positive, such as improving cardiac activity, reducing total cholesterol levels, having anti-inflammatory and antioxidant effects, regulating blood pressure, and improving blood flow [6,7,8,9,10,11,12].

Due to this duality of positive and negative effects, this integrative review analyzed the impact of pre-workout supplements on cardiovascular health in order to clarify their effects and advise users regarding their safety, particularly possible effects on the user’s hemodynamic levels.

## 2. Methods

This integrative literature review analyzed scientific studies investigating the use of pre-workout supplements and their relationship with the cardiovascular system. In order to provide greater transparency and clarity about the methods of this review, we used the systematic review protocol from the “Preferred Reporting Items for Systematic Reviews and Meta-Analyses” [13].

### 2.1. Search Strategy

The search was performed in the PubMed and Web of Science databases. The search was conducted by two independent researchers (YFB and SRSC); disagreements on the entry or exclusion of articles were resolved by a third researcher (RPV). Additionally, the reference lists of reviews were examined to identify other relevant studies. The search covered English-language articles published in the last thirteen years, from January 2010 to August 2023.

The descriptors extracted from the “Health Sciences Descriptors” (DeCS/MeSH) and keywords were combined using the Boolean operators OR and AND as follows: (“pre workout” OR “ergogenic aids” OR “supplements pre workout”) AND (“adverse effects” AND “arrhythmias” AND “ischaemic heart disease” AND “heart health” AND “heart disease”).

A second search was conducted to broaden the scope to include more recent articles and to extend the search locations. One of the other researchers (PAQI) independently performed this search. The search used the previously discussed strategies, incorporating newly published research articles and case reports from 2024.

### 2.2. Inclusion and Exclusion Criteria

Original clinical studies conducted in humans, reviews, systematic reviews, and meta-analyses were considered for inclusion. The PICOTS framework model was used to determine the inclusion and exclusion criteria. (1) Population: clinical studies in humans, of any sex, age, or level of physical conditioning (athletes or non-athletes). We did not limit the age range, in order to guarantee a greater entry of possible studies within the scope of the review. (2) Intervention: studies that evaluated the effects of multi-ingredient pre-workout supplements (MIPWs) and possible adverse cardiovascular effects as the primary outcomes. (3) Comparator: studies that used a control drink or placebo were included. (4) Outcome: studies that included the effects of MIPWs (including drink or food supplements that are commonly marketed as products to be consumed before engaging in any form of training and are characterized by a mix of nutrients that can promote positive effects on exercise performance) on cardiovascular outcomes as the primary outcome and changes in physical performance as secondary outcomes. (5) Time: intervention studies with acute or chronic effects were included in this review. The exclusion criteria included articles that were not published in journals in English, studies without complete abstracts available, studies that were not available, and studies in pre-print that have not gone through a double-blind review process. This approach guarantees a greater scope for the findings and maintains the methodological quality of the articles indexed in the body of this review. Studies that were not available in full were requested by the researchers directly from the corresponding authors through ResearchGate, a platform that connects researchers and allows them to share their research.

## 3. Results

**Figure 1:**
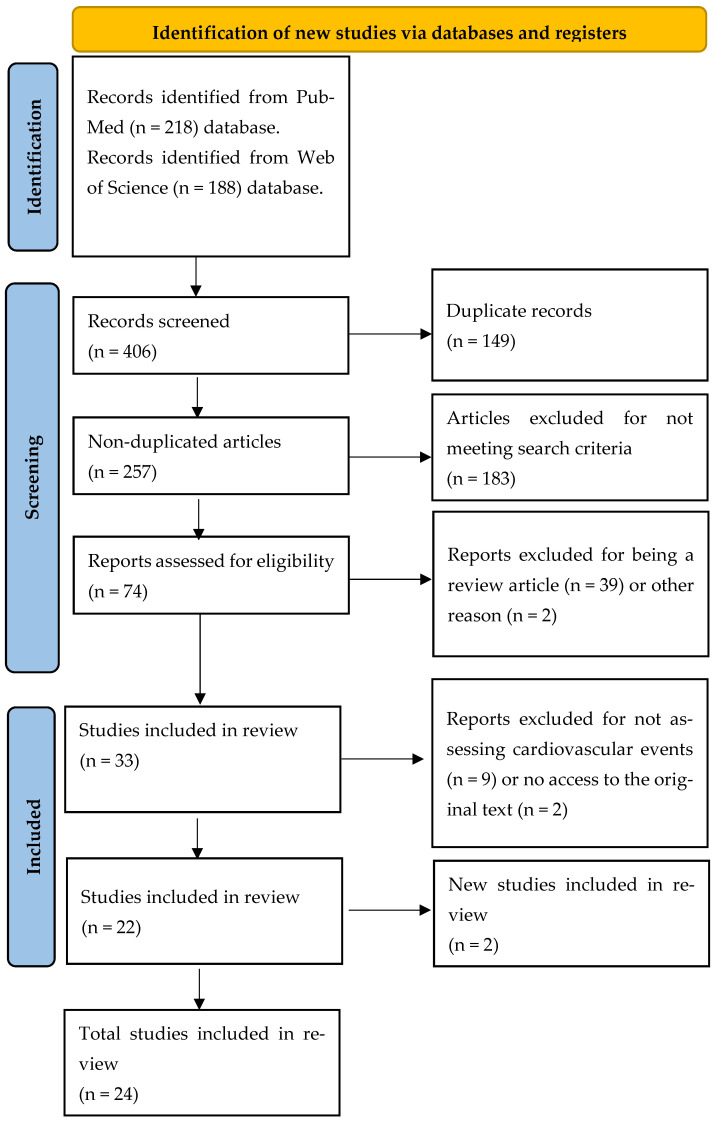
Flowchart describing the inclusion and exclusion of articles in this review according to the PRISMA protocol.

A total of 406 studies were found through the electronic and manual searches. Due to duplication, 149 studies were removed from the list. After reviewing the titles and abstracts, 224 articles were excluded, leaving 33 for the full-text analysis. Of these, two were excluded due to the inability to locate the full text, and nine did not evaluate cardiovascular markers. The results of the remaining 24 articles were synthesized into a narrative form for this integrative review. The second search found two articles that were later included (Figure 1).

### 3.1. Cardiovascular Outcomes

Among the 24 articles included, only 4 studied cardiovascular complications and hemodynamic changes, and the remaining 20 investigations on acute or chronic MIPW consumption did not find deleterious changes in cardiovascular parameters, such as systolic or diastolic blood pressure, arrhythmias, cardiac events, or adverse events. The outcomes are listed in Table 1 and Table 2.

### 3.2. Synephrine

Of the 24 articles, only 4 used an MIPW containing synephrine. In the Section 4, we will present the rationale for using this type of product and its cardiovascular effects.

### 3.3. Caffeine

Caffeine antagonizes adenosine receptors, allowing for the release of catecholamines, which alter cardiovascular responses. Its effects are augmented when co-administered with isopropylnorsynephrine or other by-products. Caffeine is one of the most common components of pre-workout supplements. Of the 24 articles, 22 reported the use of MIPWs containing caffeine, with the dosage ranging from 100 to 400 mg. For more information, see Table 1 and Table 2.

### 3.4. Other MIPW Ingredients—Creatine, Beta-Alanine, Nitric Oxide

Pre-workout supplements also commonly contain creatine and beta-alanine. Of the studies included in this review, 7 investigations used MIPWs with creatine or creatine monohydrate, 16 studies used MIPWs with the non-essential amino acid beta-alanine, and 4 studies used MIPWs containing nitric oxide. Some studies also added vitamin complexes from the B1 family to the MIPW, as well as other compounds (see Table 1 and Table 2).

### 3.5. Risk Factors, Age, Training Level, and Other Factors

Some risk factors that may contribute to adverse cardiovascular outcomes were reported in some studies, including smoking, alcohol consumption, a high body mass index, high blood pressure, and a family history of cardiovascular disease. In one case report, no pre-reported risk factors for the patient were highlighted [15]. Similarly, in another reported case, the patient had no history of cardiovascular risks. However, the study found that she was highly sensitive to caffeine, which explained her condition [16]. In contrast to these results, a case study reported a patient with a history of hypertension, smoking, and alcohol consumption, and was suffering hospital complications, which were correlated with the use of MIPW beverages [20]. The remaining articles included participants who were physically active or who were already engaged in training sessions and did not have any previous cardiovascular complications. In the 24 articles, the majority of the investigated population was young adults with an average age of between 18 and 35 years.

### 3.6. Dose and Time of Use

Most articles used MIPWs with varying doses of the ingredients, with the dose of caffeine—the most common ingredient—ranging between 100 and 300 mg. One article investigated the amounts of several ingredients used in MIPW drinks and found that the amounts of beta-alanine and caffeine were below or at the lower limit of the recommended doses for efficacy [19], while the doses of beta-alanine varied between 1 and 3 g, the creatine content was approximately 5 g, and the synephrine doses ranged between 10 and 200 mg. Some studies did not provide the doses for each ingredient. The amount of time between consuming the MIPW and engaging in any type of exercise in the studies ranged between 30 and 40 min.

## 4. Discussion

MIPWs contain a blend of ingredients designed to elicit an optimized effect on acute exercise performance and subsequent training adaptations [5,38]. These supplements typically contain ingredients such as synephrine, caffeine, creatine, beta-alanine, taurine, and nitric oxide boosters [1,4,5,14]. Some of these components are commonly associated with adverse cardiovascular effects anecdotally and, due to the increasing use of MIPW drinks, this review sought to discuss and present the current knowledge on the safety and efficacy of the use of these products and their effects on cardiovascular outcomes. Our findings corroborated the safety of MIPWs. Cardiovascular events such as palpitations and hemodynamic changes were only reported in cases with other factors affecting cardiovascular health, which could not be controlled for and prevents us from making conclusions based on these results. The following discussion explains the most common ingredients used in MIPWs.

### 4.1. Creatine

It is common to add ingredients such as creatine and beta-alanine to pre-workout supplements, especially in products that are marketed as drinks to be ingested before some activity or workout. Creatine, commonly used by bodybuilders and recreational practitioners in the gym, increases the creatine phosphate reserves in muscle tissue, increasing the energy reserves for short-term tasks and creating maximum and explosive strength. Creatine monohydrate, a non-essential energy compound synthesized by combining three amino acids (arginine, glycine, and methionine), is also found in various pre-workout supplements and is used to maintain high-energy phosphate levels during exercise. It has been shown that creatine monohydrate provides beneficial effects in physical activities, including increasing the overall average power and muscle mass development during training, and extending the time to exhaustion [39].

Creatine in safe doses (up to 5 g/day) has been shown to have considerable beneficial effects for the cardiovascular system. Creatine plays a crucial role in the production and storage of adenosine triphosphate (ATP), indirectly contributing to the maintenance of optimal cardiac function as ATP is an essential energy molecule for several bodily functions, including cardiac activity. In addition, studies suggest that creatine supplementation may have a protective effect on cardiac muscle tissue. This is due to its antioxidant properties, which help to mitigate the effects of oxidative stress, a process associated with several heart diseases, such as myocardial infarction and heart failure [7,39,40]. Our results support the use of creatine in MIPW products as it is safe and does not have hemodynamic effects. It is worth noting that, although this is not the topic of this review, it has been suggested that the use of creatine should be chronic and continuous, as its acute intake does not seem to increase adenosine triphosphate reserves. This same scenario also occurs with beta-alanine, which is discussed below.

### 4.2. β-Alanine

Beta-alanine, a non-essential amino acid, is often used as a pre-workout supplement due to its crucial role in carnosine synthesis. Carnosine is a dipeptide that acts as an intracellular pH buffer, regulating the acid–base balance in muscle cells during exercise. Thus, beta-alanine can significantly contribute to improved endurance, allowing for athletes to optimize their performance and support more intense workloads for prolonged periods [6]. Like creatine, carnosine also has anti-inflammatory and antioxidant properties; thus, beta-alanine supplementation could indirectly contribute to cardiovascular system health [10]. β-alanine in MIPW formulas does not seem to generate adverse effects on the cardiovascular system. A recent meta-analysis, based on 12 randomized clinical trials in humans, concluded that betaine supplementation, when used at doses below 4 g/day, has a positive cardiovascular effect through reducing homocysteine concentrations [6].

A study conducted in animals supplemented with β-alanine and subsequently subjected to 45 min of left coronary artery trunk occlusion showed a 57% reduction in infarct size compared to the control area. Although this effect was attributed to taurine depletion induced by the high beta-alanine intake, the possible role of carnosine and N-acetylcarnosine was not evaluated in this study [10]. These findings corroborate the safe and possibly effective use of β-alanine in MIPWs. As previously mentioned, it has been suggested that it should be used chronically. This should be investigated in future work or randomized clinical trials. However, the use of β-alanine is commonly accompanied by symptoms of paresthesia, which could make it difficult to blind clinical study participants to the presence/absence of this compound.

### 4.3. Caffeine

Caffeine, an alkaloid present in plants, is one of the most widely used stimulants worldwide and is known to increase energy, focus, and endurance, playing a significant role in improving physical performance. As a component of various pre-workouts, its ergogenic effect has been observed across a broad spectrum of sports modalities [14,17]. The mechanism of action of caffeine mainly occurs in the central nervous system, where it antagonizes adenosine receptors, a crucial neurotransmitter involved in sleep regulation and the promotion of tiredness and drowsiness. Additionally, it increases the activity of excitatory neurotransmitters such as dopamine and norepinephrine, resulting in greater alertness and vigilance [17].

In 2017, it was demonstrated that moderate caffeine intake (400–600 mg/day) is associated with a reduced risk of cardiovascular disease and can be considered a protective factor in healthy adults. Furthermore, caffeine consumption has not been consistently associated with changes in heart rate, cardiac output, electrocardiogram parameters, or heart rate variability [12]. Its combination with other ingredients, such as beta-alanine, which is common in MIPW drinks, does not appear to acutely affect heart rate parameters when consumed within the recommended dosages [37].

### 4.4. Synephrine

With the withdrawal of ephedrine from the market, the use of supplements containing synephrine, mainly obtained from bitter orange peel (*Citrus aurantium*), has become popular. Synephrine is an alternative adrenergic alkaloid to ephedrine, which indirectly potentiates the release of norepinephrine. Synephrine increases energy expenditure due to its lipolytic effect and may improve sports performance [41].

There was a comprehensive review of 30 case reports analyzing 35 patients presenting medical symptoms such as chest pain, palpitations, syncope, dizziness, and myalgia related to the use of synephrine-containing supplements for performance enhancement, with a primary emphasis on weight loss motivation. It was concluded that the use of pre-workout supplements containing synephrine may be associated with severe adverse health events, especially those related to the cardiovascular system. Synephrine, due to its similarity to ephedrine, may trigger increased blood pressure, an accelerated heart rate, arrhythmias, and stroke, especially when combined with caffeine [14], tested caffeine, synephrine, and other ingredients, including guarana, an herb containing a high dose of caffeine, yohimbine (an indoline alkaloid), deterenol combined with theophylline, and beta-phenylethylamine. Jung reported the use of isopropylnorsynephrine by a female patient through the consumption of an MIPW (which also included caffeine at 200 mg per capsule) [29]. However, it is relevant to note that synephrine appears to only manifest significant cardiovascular effects at doses exceeding 100 mg, and some studies have suggested that isolated synephrine consumption may not raise blood pressure [42,43,44].

### 4.5. Nitric Oxide and Other Ingredients

Nitric oxide is a powerful vasodilator and is important in scenarios that involve high energy expenditure and cardiovascular demands. It helps relax blood vessels, thus improving blood flow and regulating blood pressure, positively impacting cardiovascular health. It is synthesized from nitrate, which can be found in specific foods. Its effects include relaxation of blood vessels, regulation of blood pressure, and a possible improvement in exercise performance in specific populations [45]. These factors have led to the use of this compound in MIPW drinks (Table 1). In one study, a favorable effect was observed on blood lipid profiles, with a reduction in triglyceride levels and an increase in high-density lipoprotein (HDL) cholesterol levels, which are associated with a lower risk of cardiovascular diseases [7].

Taurine, or 2-aminoethanesulfonic acid, is a common component in energy drinks. Its biological mechanisms are based on its ability to conjugate bile acids, modulate Ca++ homeostasis, regulate blood pressure, and act as an antioxidant and anti-inflammatory agent [9]. In addition, taurine is essential for maintaining normal cardiac contractile function [46]. Despite elevated serum cholesterol levels in some individuals taking taurine at high concentrations, it is associated with protection against coronary heart diseases and has been used as a treatment for these conditions in Japan since 1985 [47].

Betaine (trimethylglycine) is a methylated amino acid that was first isolated from sugar beet. It has ergogenic effects in doses ranging from 500 to 9000 mg/day and can promote reductions in adiposity and/or increases in muscle mass [48]. Interestingly, betaine can also help reduce fatigue and muscle damage. It is believed to have anti-inflammatory properties, which can aid in post-workout recovery by attenuating the stress and inflammation induced by intense physical activity [48,49].

A systematic review and meta-analysis published in 2022 highlighted the significantly positive effect of betaine supplementation on total cholesterol, low-density lipoprotein (LDL) cholesterol, homocysteine, dimethylglycine, and methionine concentrations [6]. Additionally, it was observed that betaine supplementation did not negatively influence blood pressure and the serum concentrations of triglycerides, high-density lipoprotein (HDL) cholesterol, fasting glucose, C-reactive protein, and liver enzymes (alanine aminotransferase, aspartate aminotransferase, and gamma-glutamyl transferase) [6]. These parameters, when elevated, are associated with a higher risk of cardiovascular diseases.

Some pre-workout supplements have been developed to increase nitric oxide (NO) production, with the expectation that they will improve physical training performance, mainly endurance. L-citrulline and L-arginine are amino acids often found in supplements marketed as nitric oxide boosters [50]. L-citrulline is a non-essential amino acid with antioxidant properties, and is used in the urea cycle as a precursor of L-arginine which, in turn, is a precursor amino acid of nitric oxide, a substance known for its role in vasodilation and regulation of blood flow, suggesting a possible interconnection between the consumption of these supplements and the promotion of cardiovascular health [8]. Nitric oxide plays a significant role in protecting against the onset and progression of cardiovascular diseases. Its major cardioprotective roles include the regulation of blood pressure and basal vascular tone, prevention of platelet aggregation and leukocyte adhesion to the endothelium, and regulation of myocardial contractility [11].

### 4.6. Adverse Cardiovascular Outcomes

A study with 32 men and women aged between 16 and 57 years showed that the use of MIPWs causes cardiovascular anomalies. The main symptoms were chest pain (n = 11), palpitations (n = 4), syncope (n = 6), dizziness (n = 6), and myalgia (n = 4). The most common diagnoses were ischemic heart disease (n = 10), cardiac arrhythmias (n = 4), and cerebrovascular disease (n = 2), which were mainly observed after the use of pre-workouts containing synephrine [14]. Consistent with these findings, a study conducted with 63 men who regularly exercise showed that the most common adverse effects of MIPW consumption were insomnia, tremors, headache, palpitations, skin itching, and a burning sensation [17]. A more recent case study [20] reported the admission of a middle-aged man to an emergency care center with a diagnosis of rhabdomyolysis. This condition was associated with the recurrent use of an MIPW drink over the past two months containing caffeine, creatine, and nitric oxide. The study concluded that the patient exhibited electrolyte imbalances, as well as kidney and liver damage. However, no evidence of ischemia or cardiovascular injuries was observed. This review focused on cardiovascular effects, and it is notable that none were reported in this case, apart from the patient’s pre-existing hypertension, which did not appear to be exacerbated by the MIPW. The adverse effects found in the analyzed studies include a small amount of evidence from case studies, where the patients already had factors that may aggravate cardiovascular complications, such as sensitivity to caffeine, a sedentary lifestyle, a family history of cardiac complications, and a history of smoking and/or alcoholism.

### 4.7. Motivations for Using Multi-Ingredient Pre-Workout Supplements

In 2016, it was estimated that the dietary supplement industry generated an economic impact of USD 122 billion, with USD 278 billion projected for 2021. Currently, the global economic impact of the dietary supplement market is expected to expand by approximately 9% annually, potentially reaching USD 327.420.1 million by the year 2030 [51]. 

According to the “National Health and Nutrition Examination Survey” taken by the American population, approximately 50% of adults regularly consume one or more dietary supplements. Similarly, a survey conducted in 2022 by the Council for Responsible Nutrition found that 75% of respondents use nutritional supplements, with 39% reporting the use of supplements for sports purposes [52,53]. An IOC consensus statement: dietary supplements involving elite athletes highlighted that the consumption of pre-workout supplements is more prevalent in this group compared to recreational athletes, and it was observed that this pattern did not vary significantly between genders [1].

A study with 12 adult men who used pre-workout supplements acutely evaluated the resistance, strength, and power of their upper and lower limbs using cycle ergometry. There was a 9% increase in the total exercise volume and a 14% increase in the lower limb volume compared to the placebo group. These results indicate the potential use of pre-workouts to improve endurance exercise volume [54].

Another study evaluated the effects of MIPW intake in 12 physically active men in the context of a high-intensity interval exercise protocol. Pre-workout supplement intake was found to improve aerobic and anaerobic energy; compared to placebo, there was a significant increase in the number of efforts made (MIPW (41 ± 10) vs. placebo (36 ± 12), *p* = 0.0220) and in the time to exhaustion (MIPW (20.1 ± 6 min) vs. placebo (17 ± 5 min), *p* = 0.0226) [55].

The consumption of MIPWs by recreationally trained middle-aged adults for five days was shown to increase endurance and promote fat oxidation during low-intensity exercise [56]. In contrast, an analysis of acute pre-workout supplement consumption in 12 adult men during upper limb resistance training did not show improvements in performance or blood flow [22].

On the other hand, in another study, in twelve adult women using MIPW acutely and performing three treadmill running sets at 90% of their ventilatory threshold, no performance benefits related to energy metabolism were observed, but there was a reduction in effort perception [22]. In agreement with this, a study using chronic pre-workout supplements for seven weeks and involving a resistance-training program in active women found that the supplements were not effective in improving body composition and training adaptation. It is important to note that no negative hematological or metabolic changes were recorded in these women.

These findings show the rationale for the popularity of the use of these multi-ingredient beverages among amateur practitioners, recreational trainers, and those who engage in some type of training, even if there may be adverse effects (see Table 1).

### 4.8. Limitations and Future Research

This review, despite its extensive body of included articles, is not without limitations. First, only two databases were used. We recognize that this approach may have missed some relevant studies. However, we chose this approach to easily find articles that were peer-reviewed and published in English. Future research should perform a more comprehensive search. Second, despite adopting a systematic process, this was an integrative narrative review and did not use statistical methods or calculate effect sizes, which are commonly performed in meta-analyses. Finally, although the PICOTS framework was used to determine the inclusion and exclusion criteria applied in our searches, future review articles may benefit from quality analyses using tools such as the PEDRO scale [57], Black and Downs [58] scale, and risk of bias assessment tools. This review summarizes the important findings from studies on MIPWs’ effects on cardiovascular health and can be used as a resource for systematic and meta-analyses in the future. However, our findings should not be extrapolated to other populations that were not included in the scope of this investigation, such as the elderly, children, or high-performance athletes; investigations into these populations could be the focus of future research.

### 4.9. Practical Applications

Our findings corroborate that MIPW products are safe when taken in the doses recommended by the manufacturers. People already engaged in physical activities can safely take products containing caffeine, beta-alanine, synephrine, taurine, arginine, creatine, and other commonly used MIPW ingredients without significant or deleterious effects. In addition, these compounds may have a positive effect on the cardiovascular system. However, people with a history of complications from cardiovascular diseases, with a sedentary lifestyle, and/or who smoke or consume alcohol should consult doctors and specialists before taking any products.

## 5. Conclusions

MIPWs are formulated to enhance physical performance, but can also have direct effects on cardiovascular health. The most common components in these supplements, such as caffeine, creatine, beta-alanine, betaine, taurine, and nitric oxide boosters, have been associated with potential benefits for cardiovascular health.

The reviewed studies showed that pre-workout supplements may provide improvements in cardiovascular health, including reductions in triglyceride, low-density lipoprotein (LDL), and homocysteine levels, regulation of blood pressure and basal vascular tone, prevention of platelet aggregation and leukocyte adhesion to the endothelium, and regulation of myocardial contractility. These findings suggest that the use of pre-workout supplements may have positive impacts on heart and blood vessel health; however, MIPWs should be used with caution, as any abuse of MIPWs may result in the opposite effects.

## Figures and Tables

**Table 1:** Summary of results of case report and research articles.

Author/Year	Study Type	Participants	Supplement (Ingredients)	Cardiovascular and Other Reported Adverse Outcomes
De Jonge et al., 2023 [14].	Case Report	35 patients (16 man and 16 women) Age: 16–57 Healthy, trained and untrained	MIPW (synephrine: 12 to 100 mg)	Chest pains and palpitations Cardiac arrhythmia and ischemia Cerebrovascular diseases
Rodrigues Guerra et al., 2023 [15].	Case Report	1 woman Age: 35 No activity level reported	MIPW (caffeine and nitric oxide—doses not disclosed)	Tachycardia and elevated troponin levels Subclinical hyperthyroidism
Wang S.S.Y., 2020 [16].	Case Report	1 woman Age: 33 Healthy	Alpha Lean-7^®^ (caffeine, synephrine, green tea extract, and betaine) (200 mg caffeine)	Chest tightness Dyspnea and presyncope
Pilegaard et al., 2022 [17]	Observational study	63 male gym-goers Age: 15–35 Trained	MIPW (several proteins, caffeine, and green tea) (caffeine dose varied from 5 to 1323 mg per day)	Pre-workout supplements were responsible for 53% of the adverse effects reported Main cardiovascular symptom reported was palpitations Insomnia, tremors, and headaches
Knapik et al., 2022 [18]	Observational study on adverse effects in dietary supplement users in the US military service	2005 US military service members Trained and healthy	C4 Extreme^®^ C4 original^®^ (caffeine, beta-alanine, and creatine) Caffeine intake: 218 ± 2 and 167 ± 3 mg/day for men and women	Palpitations and/or increased heart rate (11%) Numbness and tingling (13%), diarrhea (5%), insomnia (5.5%), nausea/vomiting (3.5%), and tremors and seizures (1.5%)
Jagim et al., 2019 [19]	Interviews with MIPW users to assess their customs, habits, and safe usage of MIPWs	872 interviewees (636 men and 233 women) Age: 27.7 ± 8	MIPW (beta-alanine (87%; 2.0 ± 0.8 g), caffeine (86%; 254.0 ± 79.5 mg), citrulline (71%; 4.0 ± 2.5 g), tyrosine (63%; 348.0 ± 305.7 mg), taurine (51%; 1.3 ± 0.6 g), and creatine (49%; 2.1 ± 1.0 g))	Accelerated heartbeat/palpitations (23.4%) Nausea (26.6%), skin reactions (34.3%), and dizziness (14.7%)
Altaf et al., 2024 [20]	Case report	36-year-old male with hypertension and a history of renal stones Trained but not healthy	MIPW (creatine, caffeine, nitric oxide, and blast complex composed of arginine, alpha-ketoglutarate, citrulline malate, L-arginine HCL, beta-alanine, and L-norvaline) (3000 mg caffeine, creatine, and nitric oxide)	No cardiac arrhythmia, ischemia, or adverse cardiovascular events were reported, but rhabdomyolysis associated with the use of MIPWs was reported

MIPW: multi-ingredient pre-workout supplement.

**Table 2:** Summary of the results of selected clinical studies.

Author/Year	Objective of the Study	Participants	Supplement (Ingredients)	Cardiovascular and Other Reported Adverse Outcomes	Ergogenic Effects
Cameron et al., 2018 [21]	Investigation of the effect (safety and performance) of the acute consumption of pre-workouts in women undergoing resistance training	30 recreationally active women: 15 given MIPW vs. 15 given placebo Age: 21.5 ± 1.7	MusclePharm, Fitmiss™ Ignite™ (caffeine, beta-alanine, beet root, L tyrosine—doses not disclosed)	Increased diastolic blood pleasure	Increased muscular resistance, anaerobic capacity, and increased perception of focus.
Erickson et al., 2020 [22]	Evaluation of the effect of acute pre-workout intake during moderate-intensity treadmill running	12 women: 6 given MIPW vs. 6 given placebo Age: 25 ± 9 No activity level reported	Cellucor Bryan TX^®^ (caffeine (150 mg), beta-alanine (1.6 g), explosive energy blend, L-dopa, carnitine and vitamin mix)	Increased diastolic blood pleasure in supplemented group	There was no improvement in performance markers, but there was an increase in the feeling of effort.
Kedia et al., 2014 [23]	Assessment of the effect of supplements on performance and body composition during supervised resistance exercises	40 men and women: 22 given MIPW vs. 18 given placebo Age: 26 ± 5 No activity level reported	MIPW (creatine, betaine, and Dendrobium extract—doses not disclosed)	Increase in systolic and diastolic blood pressure and heart rate in the MIPW group (acute effect). These differences were not noted after six weeks. No changes in clinical and laboratory parameters (hemodynamic, biochemical, and liver enzyme parameters) in both groups	There was an improvement in the subjective feeling of focus and resistance to fatigue, but no improvement in global markers of muscle performance and body composition.
Curtis et al., 2023 [24]	Evaluation of the effect of multi-ingredient supplements on physical and mental performance	14 well-trained men and women: 7 given MIPW vs. 7 given placebo Age: 19.9 ± 1	MIPW (caffeine (250 mg), beta-alanine (2.5 g), and creatine (5 g))	Heart rate and blood pressure did not changed with supplementation	Significant improvements in attention, reaction time and measures of vigor and fatigue in the supplemented group.
Fye et al., 2021 [25]	Evaluation of the effect of multi-ingredient supplements on NCAA division athletes during a running test	11 athletes (6 men and 5 women) Age: 20 ± 2	Perform Elit™ (beet root, caffeine (150 mg), beta-alanine (3200 mg))	No difference in heart rate was observed	There was an increase in time until fatigue and lactate levels in the supplemented group.
Blake et al., 2020 [26]	Assessment of the effect of pre-workouts on blood flow and heart rate compared to the a single ingredient (caffeine) during resistance exercises	12 well-trained men: 4 given MIPW vs. 4 given pure caffeine vs. 4 given placebo Age: 22.7 ± 4	Iron Pump™ (caffeine, nitric oxide blend, vitamins) and caffeine pill (Bitartarato de colina, L-tirosina, cafeína anidra, vinpocetina (2.051 mg))	There was no change in heart rate or blood pressure No change in blood flow markers	There was no difference in perceived effort between the two groups. There was no improvement in performance in either group.
Schwarz et al., 2019 [27]	Evaluation of the safety and effectiveness of using the supplement in resistance exercises for 4 weeks	16 recreationally trained men: 8 given MIPW vs. 8 given placebo Age: 22.5 ± 3	Bang^®^ Master Blaster^®^ Pre-Workouts (caffeine (350 mg), beta-alanine (2400 mg), creatine (5000 mg), betaine (2500 mg))	Hemodynamic parameters and other blood health markers did not change after taking the supplement	Increased lean mass and greater adaptation to the use of maximum strength in resistance exercises.
Nelson et al., 2019 [28]	Investigation of the effect and safety of consuming pre-workouts in women undergoing resistance training over a seven-week period	19 recreationally active women: 10 given MIPW vs. 9 given placebo Age: 18–30	Muscle Pharm, Fitmiss™ Ignite™ (5700 mg of caffeine, beta-alanine, beet root, and L tyrosine blend)	No changes in blood pressure, heart rate, or lipid profile	No effect of supplementation on performance was observed.
Jung et al., 2017 [29]	Assessment of the effects of acute intake of supplements with and without synephrine on perception of readiness, cognitive function, and performance and health markers	50 recreationally active men and women: 25 given MIPW + synephrine vs. 25 given placebo	MIPW (beta-alanine (3 g), creatine nitrate as a salt (2 g), arginine alpha-ketoglutarate (2 g), N-acetyl-L-tyrosine (300 mg), caffeine (284 mg), Mucuna pruiriens extract standardized for 15% L-Dopa (15 mg), ascorbic acid (500 mg), niacin (60 mg), folic acid (50 mg), and methylcobalamin (70 mg) with 2 g of maltodextrin and flavoring) or a PWS with Citrus aurantium extract and synephrine (20 mg) (PWS + S)	No differences were observed in heart rate, blood pressure, electroencephalogram, and overall blood biochemical profile.	There was an improvement in the perceptions of readiness for performance and cognitive function. The effects on muscular endurance and aerobic capacity were limited or nonexistent in the groups supplemented with and without synephrine.
Jung et al., 2017 [30]	Assessment of the effects of chronic intake (8 weeks) of supplements with and without synephrine on perceived readiness, cognitive function, and performance and health markers	80 well-trained men: 27 given MIPW vs. 26 given supplement + synephrine vs. 27 given placebo Age: 22.0 ± 3	MIPW (beta-alanine (3 g), creatine nitrate as a salt (2 g), arginine alpha-ketoglutarate (2 g), N-acetyl-L-tyrosine (300 mg), caffeine (284 mg), Mucuna pruiriens extract standardized for 15% L-Dopa (15 mg), ascorbic acid (500 mg), niacin (60 mg), folic acid (50 mg), and methylcobalamin (70 mg)) or an MIPW supplemented with Citrus aurantium extract containing 20 mg of synephrine	No adverse effects reported	Increased cognitive function and performance during resistance exercises; no additional benefit from adding synephrine.
Collins et al., 2017 [31]	Evaluation of the effects of pre-workouts on performance and recovery in muscular endurance training	25 well-trained men and women Age: 23.9 ± 4	“Ready to drink” (caffeine (200 mg), β-alanine (2.1 g), arginine nitrate (1.3 g), niacin (65 mg), folic acid (325 mcg), and vitamin B12 (45 mg))	No adverse effects were reported	No differences were observed between the groups.
Vogel et al., 2015 [32]	Assessment of the safety of pre-workout supplement consumption for 28 days	34 recreationally active adult women: 18 given MIPW vs. 10 given placebo Age: 27.1 ± 5	MIPW (1 g of carbohydrate, 23 mg of calcium, and 5700 mg of a proprietary blend consisting of beta-alanine, choline bitartrate, L-tyrosine, glycine, taurine, L-carnitine, beetroot extract, hawthorn berry powder, agmatine sulfate, caffeine anhydrous, and huperzine)	Hemodynamic and biochemical markers and vital signs did not change after taking the supplement	Not evaluated.
Joy et al., 2015 [33]	Assessment of the safety of chronic use (28 days) of a pre-workout supplement	44 men and women: 14 given one dose of MIPW vs 18 given two doses of MIPW versus 12 given placebo Age: 27 ± 5	Iron Pump™ (L-arginine, nitric oxide, and caffeine—doses not disclosed)	No changes in laboratory parameters (cardiac, kidney, and liver function markers) were observed	Not evaluated.
Martin et al., 2017 [34]	Investigation of the effect of pre-training on blood flow and hyperemia during resistance training	30 recreationally active men and women: 15 given MIPW vs. 15 given placebo Age: 22.1 ± 1.3	Reckless™ (L-arginine, L-citrulline, creatine, L-norvaline, elevATP^®^, and Spectra™ (β-alanine (1600 mg) caffeine (300 mg), and creatine monohydrate (1500 mg)))	No adverse effects were reported	No differences were observed between the groups.
Kendall et al., 2014 [35]	Investigation of the safety and effectiveness of taking pre-workouts for 28 days	17 recreationally trained men: 9 given MIPW vs. 8 given placebo Age: 21 ± 4	MIPW (BCAAs (6 g), creatine (5 g), β-alanine (4 g), citrulline malate (1.5 g), and caffeine (300 mg))	No change in heart rate or blood pressure Normal liver and kidney markers in both groups	Decrease in the percentage of body fat, increase in lean mass, decrease in maximum oxygen consumption, and increase in the number of repetitions in the leg press with supplementation.
Smith et al., 2010 [36]	Evaluation of the effect of pre-workouts combined with three weeks of high-intensity interval training (HIIT) on aerobic and anaerobic performance	24 recreational athletes: 13 given MIPW + HIIT vs. 11 given HIIT control Age: 21.1 ± 1.9	Game Time^®^ (proprietary blend: 2100 milligrams of Cordyceps sinensis, arginine AKG, Kre-Alkalyn, Citrulline AKG, *Eleutherococcus senticosus*, taurine, leucine, *Rhodiola rosea*, sodium chloride, valine, isoleucine, caffeine, and whey protein concentrate)	No adverse effects were reported	There was an increase in training volume and critical speed in the supplemented group. There was no difference in the performance markers and body composition between the groups.
Martos-Arregui et al., 2024 [37]	Investigation of the acute effects of caffeine and beta-alanine administered prior to four supersets of bench presses and bench pulls on mechanical, metabolic, cardiovascular, and perceptual variables	21 young resistance-trained males Age: 23.5 ± 4.5	Caffeine alone (200 mg), beta-alanine alone (3 g), or their combination (200 mg caffeine and 3 g beta-alanine)	No adverse effects were reported	Supplement did not significantly affect any mechanical variables. Heart rate was consistent across the different groups. There was no difference in performance between the groups.

MIPW: multi-ingredient pre-workout supplement.

## Data Availability

No new data were created or analyzed in this study. Data sharing is not applicable.
